# Community pharmacists’ knowledge and perspectives of reporting adverse drug reactions in Australia: a cross-sectional survey

**DOI:** 10.1007/s11096-018-0700-2

**Published:** 2018-08-10

**Authors:** Raymond Li, Colin Curtain, Luke Bereznicki, Syed Tabish Razi Zaidi

**Affiliations:** 1 Pharmacy, School of Medicine, University of Tasmania, Hobart, Australia; 2 School of Healthcare, University of Leeds, Baines Wing, Wood House Lane, Leeds, West Yorkshire, LS2 9JT United Kingdom

**Keywords:** Adverse drug reaction, Australia, Drug safety, Pharmacist, Pharmacovigilance

## Abstract

*Background* Under-reporting of adverse drug reactions (ADRs) by healthcare professionals is prevalent worldwide. Community pharmacists are the most frequently visited healthcare professional and are well placed to document ADRs as a part of their routine practice. *Objective* To measure community pharmacists’ knowledge and perspectives towards ADR reporting and their reporting practices. *Setting* Community pharmacists in the New South Wales, Queensland, Victoria and Tasmania, Australia. *Method* A survey tool consisting of 28 items was developed, piloted and validated by a panel of expert reviewers. The final anonymised survey was distributed online to community pharmacists. Exploratory factor analysis and Cronbach’s alpha were used to measure the validity and reliability of the tool, respectively. Non-parametric statistical tests were used to analyse knowledge, perspectives and ADR reporting practices. *Main outcome measures*: Knowledge, perceived importance, enablers and barriers to reporting ADRs. *Results* The survey tool showed acceptable validity and reliability. A total of 232 respondents completed the survey. The median knowledge score was 5 out of 10 (interquartile range, 2). Less than a third of respondents (31.0%) reported sufficient knowledge and training on ADR reporting. Only 35.3% of pharmacists reported at least one ADR in the previous 12 months. Non-reporting pharmacists were more likely to report lack of time as a barrier (*P* < 0.001), conversely they were more likely to report if the practice was remunerated (*P* = 0.007). *Conclusion* Under-reporting of ADRs by community pharmacists is highly prevalent. Initiatives to educate and train them on ADR reporting and simplifying the reporting process may improve reporting practices.

## Impacts on Practice

- Overcoming barriers to ADR reporting by community pharmacists will enhance the post-marketing surveillance and the safety profile of medicines.
- Suboptimal knowledge of community pharmacists in ADR reporting presents an opportunity for this topic to be included in pharmacy curriculum.

## Introduction

Pharmacovigilance is defined as the “science and activities relating to the detection, assessment, understanding and prevention of adverse events or any other drug-related problem” [1]. The collection and reporting of adverse event information commences from the very initial stages of the drug development process through to the clinical trials, and then continuous post-marketing surveillance activities are conducted to obtain complete safety information for all medicines. Once a medicine is registered and marketed around the world, healthcare systems rely heavily on spontaneous reporting of adverse drug reactions (ADRs) to monitor the safety of medicines [2–4]. This helps regulatory agencies identify medicines that may have unidentified safety issues that were not observed during the clinical trials. In rare cases, timely identification of serious ADRs through voluntary reporting may save human lives as exemplified by the timely withdrawal of various medicines such as cerivastatin and lumiracoxib [5, 6].

Currently, the responsibility for the timely collection and reporting of safety information mainly rests with the marketing authorisation holder due to mandatory reporting requirements from regulatory agencies [2–4]. However, as consumers are more likely to report ADRs to their doctors or pharmacists rather than to the pharmaceutical industry, healthcare professionals also play a significant role in ensuring a robust pharmacovigilance system. Unfortunately, the rate of spontaneous reporting of ADRs by healthcare professionals around the world is extremely low as it is not a mandatory requirement in most countries [7, 8]. A systematic review of studies conducted in the European Union showed that the median rate of under-reporting by healthcare professionals was 94% [9]. Furthermore, a computerized surveillance study in the US showed that the rate of under-reporting was over 98% [10]. In Australia, the latest 2016 Therapeutic and Goods Administration (TGA) data show that only 4% of ADR reports they received were from general practitioners (GPs) and 5% were from community pharmacists [11]. These low rates of ADR reporting are a significant healthcare problem and can delay regulatory actions taken to remove medicines with unacceptable safety profiles from the market. A worldwide review of 462 medicines removed from the market for safety reasons showed that the median time from drug launch to drug withdrawal was 10 years [12]. These delays contribute significantly to increased healthcare costs as ADRs are a major cause of hospitalisations, morbidity and mortality with a literature review estimating that ADRs contribute to 2–12% of hospital admissions in Australia [13, 14].

Repeated calls for mandatory ADR reporting requirements for healthcare professionals have been made as patients are more likely to discuss or potentially report a problem with their medications to those who initially dispensed or prescribed them [15]. In Canada, the Protecting Canadians from Unsafe Drugs Act was introduced in November 2014 to enforce mandatory reporting of serious ADRs by healthcare institutions [16]. In Australia, the Consumers Health Forum in their submission to the 2015 Sansom review of Australian Medicines and Medical Devices regulations also argues for mandatory requirements for doctors and pharmacists to report ADRs [15]. Furthermore, the TGA is introducing a black triangle scheme in 2018 to alert healthcare professionals and consumers that a new medicine is available and to request they report ADRs associated with that medicine [17]. These measures would enhance existing pharmacovigilance systems to collect more safety information and this allows for faster action to be implemented by regulatory agencies if necessary. Doctors and pharmacists are also in the best position to report ADRs as they have frequent interactions with patients across all therapeutic areas and would therefore be an invaluable source of safety information.

International studies showed that healthcare professionals have very limited knowledge of pharmacovigilance and their perspectives towards ADRs play a strong role in influencing their reporting rates [18–23]. This relationship is clearly detailed in Suyagh et al. and Xu et al. which showed that perspectives of indifference, lack of remuneration, competing workplace priorities and dissatisfaction with reporting methods strongly impacted the ADR reporting rates [18, 20]. There is also evidence to show a relationship between ADR knowledge and reporting rates, with Herdeiro et al. identifying a 5.9 fold increase in ADR reporting after pharmacists were provided with a one hour educational session on pharmacovigilance [24]. Despite the wealth of international literature, limited data exist on the practice of ADR reporting by the Australian doctors and pharmacists. The only two Australian studies were conducted in acute care settings and as such, the perspectives of Australian community pharmacists on the topic of ADR reporting is lacking [25, 26]. It is important to note that the perspectives of community pharmacists have not been investigated to date, and it is vital that this gap is addressed as they are usually the first point of contact regarding medication related issues, as well as being the most frequently visited healthcare professional in Australia [27]. This is reinforced by the fact that over 280 million prescriptions were dispensed through the Pharmaceutical Benefits Scheme over the 2016/2017 financial year in Australia [28].

## Aim of the study

This study investigated community pharmacists’ understanding of pharmacovigilance, their perspectives towards reporting ADRs, and their actual practice of reporting ADRs in the primary care setting.

## Ethics approval

Approval was obtained from the Tasmanian Health and Medical Human Research Ethics Committee with reference H0016219.

## Methods

### Development of the survey tool

Given that there have been no studies of this type conducted in Australia, a pharmacovigilance survey tool was needed. Based on a review of the literature, we were interested in 3 specific domains namely, knowledge of pharmacovigilance, perspectives, and practices of ADR reporting. An item pool for each of these domains was generated based on the available literature, with the question selection based on consultations with senior pharmacists from the investigators’ network who were known to have an interest in this topic and practising in various settings. The draft tool underwent extensive testing and review by industry pharmacists (*n* = 5), community pharmacists (*n* = 6), hospital pharmacists (*n* = 9) and pharmacy academics (*n* = 5). The questions in the survey tool were then adjusted based on any qualitative feedback provided by the respondents and the results of the internal consistency measure. The final survey tool consisted of 3 sections; the knowledge section consisted of 10 multiple choice questions with a single correct answer, the perspectives section included 13 Likert item questions (5 point item), and the practice related questions included 4 multi-choice questions and 1 free text question. The survey tool is shown as “Appendix”.

### Sample size and recruitment of respondents

The Pharmacy Board of Australia’s registration statistics for December 2016 showed that there were 27,452 pharmacists with general registration in Australia [29]. Of these, 21,429 pharmacists were practising in the eastern states of Australia. According to the Department of Health National Health Workforce Data for 2016, there was 15,257 pharmacists working in community pharmacy [30]. Based on a confidence interval of 95% with a confidence level of +/−5%, we estimated a sample size of 375 pharmacists to represent the population [31]. Community pharmacists practising in New South Wales, Queensland, Victoria and Tasmania (eastern states of Australia) were invited to participate in this survey by email to community pharmacies, social media posts (Early Career Pharmacists Facebook page, Sydney Locum Pharmacists Facebook page, Locum Pharmacists Brisbane Facebook page, Pharmacist Locums—Melbourne Facebook page, Locum Pharmacists—Tasmania Facebook page, and Rural Pharmacy Locums Australia Facebook page), and the Australia Journal of Pharmacy discussion forum. The participant information sheet was accessible to all respondents and participation was voluntary. Completion of the survey was considered as implied consent. A single reminder message was posted on the above-mentioned forums at week 3 of the study.

### Statistical analysis

Statistical analyses of the main survey data were performed using IBM SPSS (version 24.0) with significance levels set at *P* ≤ 0.05. Non-parametric tests were used for comparing knowledge scores, perspectives, and ADR reporting practices across the different pharmacist demographics (Mann-Whitney U test for 2 groups and Kruskal Wallis for more than 2 groups). Exploratory factor analysis (principle axis factoring) was performed to simplify the number of variables into distinct factors with a non-orthogonal rotation (direct oblimin with Kaiser Normalisation) applied after extraction [32]. Negatively worded statements were reverse coded and reliabilities of each factor were measured by Cronbach’s alpha. Chi square and independent samples t test were used to compare the differences in the perceptions of pharmacists who have seen and reported at least one ADR and those who have seen but not reported any ADR to the TGA. Variables showing a p value of less or equal to 0.1 were included in a logistic regression analysis.

## Results

**Fig. 1:**
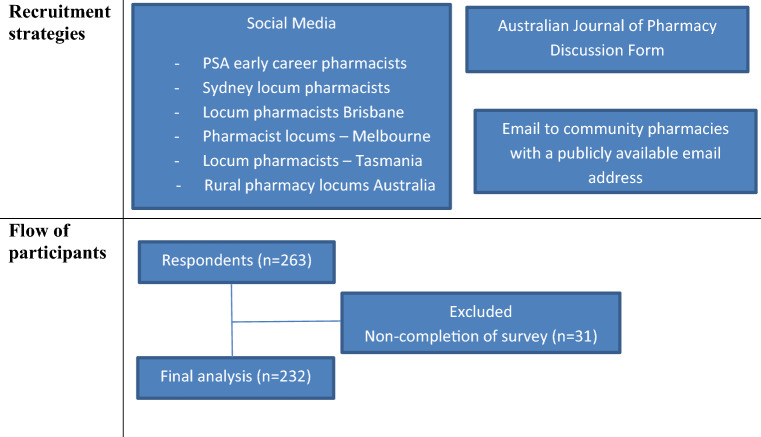
Flowchart of recruitment of participants from New South Wales, Queensland, Victoria, and Tasmania

From January to February 2017, a total of 263 surveys were collected and 232 were included in the final data analyses based on full completion of the survey (Fig. 1). Of the 31 surveys that were excluded, the respondents only provided information on their demographics with minimal completion of the questions in the actual survey, and therefore this data could not be used for analysis. The demographic details of the community pharmacists are summarised in Table 1.

**Table 1:** Respondent demographics

Variables	Respondents, *n* = 232	%
*State*		
NSW	126	54.3
QLD	47	20.3
VIC	54	23.3
TAS	5	2.1
*Hours worked per week*		
< 20 h	13	5.6
20–40 h	102	44.0
> 40 h	117	50.4
*Years of registration*		
5 years or less	80	34.5
6–9 years	69	29.7
10 years or more	83	35.8
*Professional memberships*^a^		
Yes	125	53.9
No	107	46.1
*Highest level of pharmacy qualification*		
Bachelors	154	66.4
Post-graduate^b^	78	33.6

^a^Professional memberships include Pharmaceutical Society of Australia, Pharmacy Guild of Australia, Australian college of Pharmacy, Society of Hospital Pharmacists Australia, and Australian Association of Consultant Pharmacy

^b^Post-graduate degrees include honours, graduate certificate/diploma, masters and doctorate (Ph.D.) in pharmacy

### Knowledge

The median knowledge score was 5 out of 10 (interquartile range 4–6, range 1–9). There were no significant differences in the knowledge scores of pharmacists based on hours worked per week, number of years of registration, having a professional membership, or highest level of pharmacy qualification obtained. However, there was a weak correlation between higher knowledge scores and the longer a pharmacist was registered (Spearman’s Rho 0.131, *P* = 0.047).

There were 81.9% (*n* = 190) of respondents who were able to define pharmacovigilance though only 13.8% (*n* = 32) of them correctly identified examples of an adverse event. Despite the fact that the majority of respondents (84.9%, *n* = 197) knew the type of ADRs that the TGA wants healthcare professionals to report, only 3.0% (*n* = 7) of them were able to identify the office responsible for the collection and analysis of ADRs at the TGA. Almost two thirds of respondents (60.8%, *n* = 141) were able to differentiate between an adverse event and an ADR. Conversely, less than half of the respondents correctly identified what the TGA considers as a serious ADR (8.6%, *n* = 20), the mode of communication between TGA and healthcare professionals (40.5%, *n* = 94), and the common safety issues involving medicines on the market (25.4%, *n* = 59).

### Perceived importance, facilitators and barriers of ADR reporting

The factor analysis of the perceptions section yielded a 3 factor solution based on Eigenvalues of greater than 1 explaining more than half of the total variance (Table 2). The item ‘I currently have sufficient knowledge and training on how to report ADRs’ was removed as the self-reporting of knowledge was considered a separate construct. The following section will present the results of each factor separately.

**Table 2:** Pharmacist perspectives towards reporting ADRs—factor analysis

Item	Loadings		
	Factor 1	Factor 2	Factor 3
Q11. Reporting ADRs is important for patient care	**0.590**	− 0.138	− 0.348
Q12. Reporting ADRs should be mandatory for community pharmacists	**0.542**	− 0.254	− 0.226
Q14. Pharmacovigilance should be taught in the undergraduate pharmacy programs at universities	**0.629**	− 0.099	− 0.300
Q15. Professional bodies (e.g. PSA) should organise workshops or training sessions to cover the importance of ADR reporting	**0.803**	− 0.100	− 0.354
Q18. I have a professional obligation to report ADRs	**0.728**	− 0.349	− 0.47
Q13. I don’t have the time to report ADRs as part of my professional practice	− 0.124	**0.603**	− 0.129
Q17. I fear that there may be legal repercussions if I report an ADR to the TGA	− 0.264	**0.427**	0.243
Q20. I would be encouraged to report more ADRs if it was remunerated	0.088	**0.490**	− 0.425
^a^Q19. There are no results or actions taken based on ADRs that I report	− 0.062	**0.235**	0.017
Q21. I would be encouraged to report more ADRs if there is a reminder in my dispensing software	0.521	0.041	**− 0.646**
Q22. I would be encouraged to report more ADRs if patient information is automatically populated from the dispensing software into an ADR report ready for submission	0.482	− 0.036	**− 0.936**
Q23. I would be encouraged to report more ADRs if general education is provided on the importance of pharmacovigilance	0.584	0.059	**− 0.618**
Eigenvalues	4.017	1.714	1.085
% of variance explained	33.5	14.3	9.0
Total % of variance explained			**56.8**

*Factor 1* Perceived importance of reporting ADRs. *Factor 2* Barriers to reporting ADRs. *Factor 3* Facilitators of reporting ADRs

^a^Was not classified into any factor due to inadequate loading

Highest loadings for a given item is in bold

#### Perceived importance of ADR reporting

Nearly all respondents (97.0%, *n* = 225) believed that reporting ADRs is important for patient care and the majority (93.1%, *n* = 216) felt that they are obliged to report ADRs to the relevant authority, the TGA (Table 3). More than two thirds of pharmacists (72.0%, *n* = 167) also believed that reporting ADRs should be mandatory. Most respondents believed that pharmacovigilance should be included in the undergraduate pharmacy curriculum (95.3%, *n* = 221) and professional bodies should include it in their continuous professional development activities (89.7%, *n* = 207). The factor analysis showed that all the above statements had very high loadings with acceptable reliability (Cronbach’s alpha 0.778) which appropriately explains the perceived importance of ADR reporting.

**Table 3:** Pharmacist perspectives towards reporting ADRs (%)

Question	Strongly disagree (%)	Disagree (%)	Neutral (%)	Agree (%)	Strongly agree (%)
Q11. Reporting ADRs is important for patient care	0.9	0	2.2	30.6	66.4
Q12. Reporting ADRs should be mandatory for community pharmacists	0.9	7.8	19.4	47.0	25.0
Q13. I don’t have the time to report ADRs as part of my professional practice	9.5	19.8	27.2	34.9	8.6
Q14. Pharmacovigilance should be taught in the undergraduate pharmacy programs at universities	0.9	0	3.9	52.6	42.7
Q15. Professional bodies (e.g. PSA) should organise workshops or training sessions to cover the importance of ADR reporting	0.4	1.3	8.6	55.2	34.5
Q16. I currently have sufficient knowledge and training on how to report ADRs	6.0	27.2	35.8	26.3	4.7
Q17. I fear that there may be legal repercussions if I report an ADR to the TGA	33.2	51.7	11.2	3.4	0.4
Q18. I have a professional obligation to report ADRs	0.4	0.4	6.0	56.0	37.1
Q19. There are no results or actions taken based on ADRs that I report	6.9	30.6	40.1	20.7	1.7
Q20. I would be encouraged to report more ADRs if it was remunerated	3.0	9.5	22.8	34.9	29.7
Q21. I would be encouraged to report more ADRs if there is a reminder in my dispensing software	1.7	3.9	9.5	44.4	40.5
Q22. I would be encouraged to report more ADRs if patient information is automatically populated from the dispensing software into an ADR report ready for submission	0.4	1.3	6.5	42.2	49.6
Q23. I would be encouraged to report more ADRs if general education is provided on the importance of pharmacovigilance	0.4	1.3	9.1	56.0	33.2

#### Facilitators of ADR reporting

Having patient information automatically populated from the dispensing software into an ADR report ready for submission was the most popular way to encourage pharmacists to report ADRs with 91.8% (*n* = 213) of respondents agreeing with this statement. Other factors such as providing general education on pharmacovigilance and setting a reminder in the dispensing software were also seen as effective measures with 89% (*n* = 207) and 85% (*n* = 197) of respondents agreeing with these proposals, respectively. These three items also had very high factor loadings together with acceptable reliability (Cronbach’s alpha 0.792) and was labelled as facilitators of ADR reporting.

#### Barriers to ADR reporting

There were 43.5% (*n* = 101) of respondents who agreed with the statement “I don’t have the time to report ADRs as part of my professional practice” whilst 29.3% (*n* = 68) of respondents disagreed. Approximately 65% (*n* = 150) of respondents also agreed that remuneration would encourage them to report ADRs and this shows that the current lack of remuneration for reporting ADRs in Australia is a substantial barrier. There was also a moderate correlation between not having the time to report ADRs and being encouraged to report ADRs if reporting was remunerated (Spearman’s Rho 0.364, *P* < 0.001). Furthermore, there were 22.4% (*n* = 52) of respondents who agreed with the statement “There are no results or actions taken based on ADRs I report”. Over 84% (*n* = 197) of respondents also disagreed with the statement “I fear that there may be legal repercussions if I report an ADR to the TGA”. The factor analysis showed that the items above had high loadings and described the barriers to reporting ADRs. However, the reliability of this factor was low (Cronbach’s alpha 0.488).

### ADR reporting and variables associated with reporting practices

Only 35.3% (*n* = 82) of pharmacists have reported at least one ADR to the TGA in the previous 12 months, even though 88.4% (*n* = 205) of pharmacists encountered an ADR in a patient and 65.9% (*n* = 153) record ADRs as part of a clinical intervention at least once a month. Independent samples t test showed that there was a significant difference between pharmacists who have reported ADRs versus those who have never reported based on their hours worked per week (1.52 vs. 1.33, *P* = 0.024). However, Chi squared test revealed that there was no significant relationship between the number of hours worked and the reporting of ADRs (*P* = 0.058). As evident from Table 4, the logistic regression showed that the number of hours worked per week was a significant predictor for whether pharmacists reported ADRs or not (OR 4.32, 95% CI 1.22–15.16). Furthermore, barriers to ADR reporting was also a significant predictor of whether pharmacists reported ADRs (OR 1.43, 95% CI 1.19–1.71). All other variables assessed were not significant predictors.

Respondents also provided various suggestions that may encourage pharmacists to report more ADRs as qualitative comments. These includes: making reporting a habit starting within university education, commencing an educational advertising campaign in this area, adding a prompt to report ADRs during online recording of clinical interventions, adding ADR reporting as a separate reimbursable cognitive activity, and receiving feedback on ADR reports that pharmacists had made to see what impact the reports had.

## Discussion

Underreporting of ADRs is a widespread problem around the world and the voluntary nature of reporting is not helping the situation [33]. A number of ADR presentations in acute care may be attributable to a patient’s current illness and association with a suspected drug may not be clear enough to encourage prompt reporting [34]. Community pharmacists in Australia are well placed to collect, and report ADRs to the regulatory authority as they are the publics’ most frequently visited healthcare professional [27]. However, there is a clear gap in our understanding of their knowledge and perspectives towards ADR reporting with only one third of respondents considering themselves knowledgeable and trained in ADR reporting. Therefore, this ‘first of its kind’ survey development and piloting study was conducted to investigate this missing information. The newly developed pharmacovigilance survey tool was found to be a reliable and valid measure of the knowledge, perspectives and practices of reporting ADRs in Australia.

We showed that community pharmacists’ knowledge of pharmacovigilance in Australia is limited. Knowledge scores were not higher in respondents with post-graduate qualifications or those with more years of experience indicating that pharmacists did not receive adequate education as part of their continuing professional development. This is an important unmet need and is supported by the finding that almost all respondents agreed that pharmacovigilance should be included into the curricula of undergraduate pharmacy programs and that professional organisations should organise workshops to highlight the importance of reporting ADRs. There have been some efforts to draw healthcare professional awareness to the importance of reporting ADRs with the TGA and the National Prescribing Service recently creating 2 online continuing education modules in 2014 to explain the importance of reporting ADRs and how to build reporting into practice [35]. Despite this, there was still no increase in the number of ADRs reported to the TGA by healthcare professionals in 2016 [11] and therefore, additional activities to increase awareness and address reporting barriers must be considered.

We found that only 35% of community pharmacists have reported at least one ADR to the TGA in the previous 12 months and this low reporting rate is supported by TGA data showing that only 903 ADRs were reported by community pharmacists out of a total of approximately 17,000 case reports (5.3%) in 2016 [11]. This may be attributed to the limited pharmacovigilance knowledge of pharmacists with only a third agreeing that they have sufficient knowledge and training on this topic. The low ADR reporting rates are also consistent with studies conducted across Europe, Asia and Africa, which showed reporting rates of between 14 and 44% to their respective national ADR reporting centres [18–23]. This large variance in ADR reporting rates may be attributed to the different working environments and challenges that community pharmacists face in different countries. For example, a study in Jordan showed that 30% of pharmacists feared legal liability when reporting ADRs compared to only 4% in this study [20]. A study in Iran showed that only 26% of pharmacists believed reporting ADRs is a professional obligation compared to 93% in this study [22]. However, all studies concluded that providing education and training in pharmacovigilance is essential to improve awareness, knowledge and ADR reporting rates.

Non-reporting community pharmacists indicated that lack of time was the most significant barrier to reporting ADRs in Australia. This was expected, as community pharmacists are now providing a number of professional services such as MedsChecks Program, dose administration aids, home medicines reviews, and clinical interventions in addition to their traditional role of dispensing and supplying medicines [36]. However, we also showed that community pharmacists who have reported ADRs to the TGA did not perceive time as a barrier and this suggests that it is the underlying perspectives of the individual pharmacist that affects how they allocate time to perform ADR reporting as part of their professional practice. Despite this, the concern about lack of time for non-reporting pharmacists should not be disregarded and existing processes for ADR reporting can be further simplified. Therefore, one of the priorities to improve ADR reporting rates by community pharmacists would be to shorten the time it takes to complete the process. This can be achieved by making the reporting forms more accessible, electronic, and utilise any auto-population features available from existing information in the dispensing software. In June 2014, a pharmacy software vendor Guildlink created an Adverse Events Recording module into community pharmacy dispensing software that integrates directly into the TGA ADR web service [37]. This allows community pharmacists to report ADRs directly to the TGA via their dispensing software instead of having to manually complete a separate ADR reporting form. By September 2014, the TGA received 254 reports from community pharmacists via this GuildLink portal and the number of ADRs reported by community pharmacists in the first three quarters of 2014 was almost as high as the total number of reports received from community pharmacists for the entire year in 2013 [38]. It suggests these measures to simplify the ADR reporting process were well received and further initiatives are required to increase the volume of ADRs reported by healthcare professionals. However, it is also important to highlight that ADR reporting rates by pharmacists fell again in 2015 indicating that constant reminders are necessary to encourage and maintain higher ADR reporting rates.

### Limitations and future research

One of the key limitations for this study is that the sample size of 375 community pharmacists was not reached, indicating that these results may not be representative of the entire group of community pharmacists registered in Australia. The number of pharmacists working less than 20 h was also significantly under-represented at only 5.6% of our sample compared to 13.9% in the wider community pharmacist population [30]. Secondly, there is the impact of social desirability bias from the pharmacist respondents to consider [39]. Community pharmacists may feel guilty for not reporting ADRs as often as they should as part of their professional practice and the ADR reporting frequencies recorded in this survey may be inflated. Some pharmacists may have provided ‘socially desirable’ responses about their perspectives towards the importance of reporting ADRs. However, the use of self-administered anonymised surveys would have reduced the impact of this bias.

Further research can be conducted to investigate the perspectives of other healthcare professional groups such as GPs, nurses, and hospital staff towards reporting ADRs in Australia. This may focus on looking at specific interventional strategies that address the barriers towards reporting ADRs and investigate the effectiveness of these interventions. Research can be undertaken to examine the impact of pharmacovigilance educational programs into changing the perspectives, and practice of reporting ADRs. For example, if pharmacists were provided with information on why ADR reporting is so important, they may change their perspectives and allocate more of their time to report ADRs as part of their professional practice.

**Table 4:** Variables associated with adverse drug reaction reporting in community pharmacists

Variables	Adjusted OR (95% CI)
Perceived importance of ADR reporting	1.04 (0.93–1.16)
Enablers of ADR reporting	1.02 (0.85–1.24)
Barriers to ADR reporting	1.43 (1.19–1.71)
Total marks	1.05 (0.83–1.32)
21–40 h worked per week	4.30 (1.22–15.16)
> 40 h worked per week	1.48 (0.81–2.70)

## Conclusion

The ADR reporting rate by community pharmacists in Australia is low even though the vast majority believe they have a professional obligation to report. The most significant barrier to reporting ADRs is lack of time and this can be addressed by simplifying the current reporting procedures and utilising auto-population features from existing software. This is reinforced by the fact that the reported barriers and the number of hours worked per week by community pharmacists are significant predictors of their rate of ADR reporting in Australia.

## Appendix

### Pharmacovigilance Survey Tool

#### Knowledge questions

1. What is the definition of pharmacovigilance?
  - The detection, assessment, understanding, and prevention of adverse reactions*
  - The reporting of adverse reactions
  - The science of improving the safety profile of a medicinal product
  - The science of weighing the benefit risk profile of a medicinal product
  - I do not know
  2. Which of the following is an example of an adverse event?
    - A patient taking Drug X was hospitalised for a stroke. The hospital doctor believes Drug X did not cause the stroke
    - A patient taking Drug X experienced a rash. The rash was believed to have been caused by Drug X
    - A patient taking Drug X said it didn’t work for him. He did not experience any side effects
    - All of the above*
    - I do not know
  3. What type of adverse reactions does the TGA want healthcare professionals to report in Australia?
    - Serious and unexpected adverse reactions
    - Non-serious adverse reactions
    - Expected adverse reactions
    - All adverse reactions regardless of seriousness and expectedness*
    - I do not know
  4. In Australia, which group at the TGA is currently responsible for the collection and monitoring of ADRs
    - Adverse Drug Reaction Advisory Committee (ADRAC)
    - Office of Product Review (OPR)
    - Pharmacovigilance and Special Access Branch (PSAB)*
    - Australian Drug Evaluation Committee (ADEC)
    - I do not know
  5. The TGA accepts ADR reports from:
    - Consumers and patients only
    - Healthcare professionals only
    - Hospital networks only
    - All of the above*
    - I do not know
  6. How does the TGA communicate important or new ADR information that have been confirmed?
    - TGA Early Warning System – monitoring communications
    - TGA Medicines Safety Update bulletins*
    - TGA Risk Management plan
    - All of the above
    - I do not know
  7. At which stage is the most adverse event information collected about a drug?
    - Phase I clinical trials
    - Phase II clinical trials
    - Phase III clinical trials
    - Phase IV clinical trials and post marketing surveillance*
    - I do not know
  8. Please consider the following 3 statements. Choose the CORRECT answer
    - The terms adverse event and ADR are synonymous and can be used interchangeably
    - An adverse event is considered as anything medically untoward that happens and there does not need to be an established causal relationship between the adverse event and the drug
    - ADRs are collected during clinical trials, whilst adverse events are collected during post marketing surveillance activities
    - Only statement I is correct
    - Only statement II is correct*
    - Only statements I and II are correct
    - Only statements II and III are correct
    - I do not know
  9. Which of the following scenarios would NOT always be considered as a serious adverse event by the TGA?
    - An adverse event that results in hospitalisation
    - An adverse event that is severe*
    - An adverse event that results in congenital anomaly/birth defect
    - An adverse event that was considered to be serious by a healthcare professional
    - I do not know
  10. What is the most common safety reason for withdrawing a drug from the market?
    - Hepatotoxicity*
    - Cardiotoxicity
    - Carcinogenicity
    - Nephrotoxicity
    - I do not know

*Denotes correct answer at the time of publication

#### Pharmacist practice related questions

24. How often do you see ADRs in patients?
  - At least once a week
  - At least once a month
  - At least once a year
  - Never
  25. How often do you record ADRs as part of your clinical interventions?
    - At least once a week
    - At least once a month
    - At least once a year
    - Never
  26. What is the most common method that you use to report an ADR to TGA?
    - Phone
    - Fax
    - Email
    - TGA ADR blue card
    - TGA online reporting portal
    - I have not reported any ADRs to the TGA
  27. What is the most common method that you use to report an ADR to TGA?
    - Phone
    - Fax
    - Email
    - TGA ADR blue card
    - TGA online reporting portal
    - I have not reported any ADRs to the TGA
  28. Finally, do you have any suggestions on what would encourage you to report more ADRs? (free text field)
